# Leptin and Physical Activity in Adult Patients with Anorexia Nervosa: Failure to Demonstrate a Simple Linear Association

**DOI:** 10.3390/nu9111210

**Published:** 2017-11-03

**Authors:** Andreas Stengel, Verena Haas, Ulf Elbelt, Christoph U. Correll, Matthias Rose, Tobias Hofmann

**Affiliations:** 1 Center for Internal Medicine and Dermatology, Department of Psychosomatic Medicine, Charité-Universitätsmedizin Berlin, 12200 Berlin, Germany; Matthias.rose@charite.de (M.R.); tobias.hofmann@charite.de (T.H.); 2 Department of Psychosomatic Medicine and Psychotherapy, Medical University Hospital Tübingen, 72076 Tübingen, Germany; 3 Feinstein Institute for Medical Research at Hofstra/Northwell, Islandia, NY 11749, USA; ccorrell@northwell.edu; 4 Center for Internal Medicine with Gastroenterology and Nephrology, Division for Endocrinology, Diabetes and Nutrition, Charité-Universitätsmedizin Berlin, 12200 Berlin, Germany; ulf.elbelt@charite.de; 5 Donald and Barbara Zucker School of Medicine at Hofstra/Northwell, Hempstead, NY 11549, USA; 6 Department of Psychiatry, The Zucker Hillside Hospital, Glen Oaks, NY 11004, USA

**Keywords:** accelerometry, eating disorders, motor restlessness, semistarvation-induced hyperactivity

## Abstract

High physical activity (PA) in patients with anorexia nervosa (AN) is hypothesized to be, at least in part, a consequence of hypoleptinemia. However, most studies on the association of leptin and PA in AN were performed in adolescents or young adults, and PA was generally measured with subjective tools. We aimed to explore the association of leptin and PA in adults with AN using an objective technique to quantify PA. Using a cross-sectional, observational design, we analyzed body fat (bioelectrical impedance), PA (accelerometry, SenseWear™ armband) and plasma leptin (ELISA) in 61 women with AN (median age: 25 years, range: 18–52 years; median BMI: 14.8 ± 2.0 kg/m^2^) at the start of hospitalization. Results indicated a mean step count per day of 12,841 ± 6408 (range: 3956–37,750). Leptin was closely associated with BMI and body fat (ρ = 0.508 and ρ = 0.669, *p* < 0.001), but not with steps (ρ = 0.015, *p* = 0.908). Moreover, no significant association was observed between BMI and steps (ρ = 0.189, *p* = 0.146). In conclusion, there was no simple, linear association of leptin and PA, highlighting the need for more complex and non-linear models to analyze the association of leptin and PA in adults with AN in future studies.

## 1. Introduction

High levels of physical activity (PA) are frequently observed in patients with anorexia nervosa (AN) [1]. For a long time, increased PA in AN had merely been regarded as the patients’ deliberate strategy to lose weight. However, when asked, many patients themselves experience their desire to move as “foreign” or “awkward”, some even as “frightening”, and they describe their restlessness as “difficult to suppress” [2]. In 1994, the discovery of the adipocyte-derived hormone leptin led to a major advancement in the understanding of biological pathways involved in the regulation of energy homeostasis and body weight [3]. Being synthesized in adipocytes, leptin levels decline as a consequence of reduced body fat. Accordingly, leptin levels in patients with AN are low [4,5]. In 2000, a key experiment was published, demonstrating that food restriction in an activity-based AN rat model led to increased PA by 300% [6]. A subsequent study reported that leptin administration suppressed this hyperactivity [7]. Despite the fact that animal data have to be translated with great caution to humans, from an evolutionary perspective, an increased drive for PA that would facilitate finding food at times of food shortage might have been useful for survival. Moreover, reduced leptin signaling has been linked with an increased rewarding aspect of running [8].

Based on these findings, a biological influence on altered PA has been hypothesized for AN patients. A number of human studies explored the association of leptin and PA in adolescent/adult subjects with AN; however, results have been conflicting, showing positive linear [9], U-shaped [10], inverted U-shaped [11], negative linear [6,12,13] or a lack of association [14]. So far, most clinical studies on the relationship between leptin and PA in AN were conducted in adolescents and young adults. One prior study found an association of leptin and PA only in adolescents, but not in adults with AN [10]. Therefore, the question arises whether a potential association between leptin levels and PA persists over time in adult AN patients.

The aims of the current study were to investigate whether there is an association of leptin and PA in a large adult patient group diagnosed with AN using an objective technique to measure PA and to study whether age, BMI, body fat and duration of illness are significant moderators of the association of leptin and PA. In contrast to the prevailing view that hypoleptinemia is associated with high PA in AN, we hypothesized that there is a lack of a simple association in adults with AN because of the conflicting results reported before and that age, BMI, body fat and/or duration of illness have an effect on the relationship between leptin and PA. Since AN is more prevalent in females compared to males [15], only female subjects were enrolled in the present study.

## 2. Materials and Methods 

### 2.1. Design

This was a cross-sectional, observational cohort study. The primary outcome was the association of leptin and PA, and the secondary outcome was a range of potential moderators of this association.

### 2.2. Study Population

We enrolled 61 women with AN, who were recruited at the initiation of inpatient treatment at the Department of Psychosomatic Medicine between 2012 and 2017. Patient inclusion criteria were a diagnosis of AN according to ICD-10 (International Statistical Classification of Disease and Related Health Problems of the World Health Organization, 10th revision) [16] of either typical or atypical AN. In contrast to typical AN, the subtype of atypical AN was defined as a disorder meeting most criteria of the typical type (body mass index (BMI) below 17.5 kg/m^2^, self-induced weight loss, body image distortion and endocrine disorder most often reflected by secondary amenorrhea) without fulfilling all key symptoms (e.g., BMI or amenorrhea), as described previously [17]. Patients suffering from the restrictive subtype of AN reduce weight only by dieting and in many cases by increased PA, while the purging subtype is additionally characterized by recurrent episodes of binge eating or purging behaviors, such as self-induced vomiting or misuse of laxatives. In addition to typical or atypical AN, all patients were classified with either the restrictive or purging subtype. All patients were Caucasian since ethnic variations in leptin levels have been described before [18,19]. Exclusion criteria were: pregnancy or psychotic disorder, or any major further somatic comorbidity such as malignoma or chronic inflammatory disease.

### 2.3. Ethics

The study was approved by the institutional ethics committee of the Charité-Universitätsmedizin Berlin (Protocol Number EA1/114/10), and all investigations were conducted according to the Declaration of Helsinki. All participants gave written informed consent.

### 2.4. Clinical Data

Duration of illness and current medication treatment were assessed at admission by taking the medical history of the patients. Smoking status was obtained by patient interview as part of the PA assessment using the SenseWear™ armband (SenseWear™ PRO3 armband; BodyMedia, Inc., Pittsburgh, PA, USA).

### 2.5. Body Composition

Whole-body impedance (resistance, reactance and phase angle) was performed using bioelectrical impedance analysis (BIA) on the first day of PA assessment. Data were collected using Nutriguard-M (Data Input, Darmstadt, Germany; electrodes: Bianostic-AT, Data Input). BIA measurements were standardized according to the manufacturer’s recommendations. Body fat was calculated by subtracting predicted fat-free mass from body weight with the use of the sex-specific equation [20].

### 2.6. Physical Activity

Accelerometric measurement of PA took place 4 ± 2 (range: 1–11) days after admission. At the time of measurement, patients had no restrictions with regards to their daily PA. PA was assessed using a portable armband (SenseWear™) in combination with the manufacturer’s specific software (SenseWear™ Professional, Version 8.1, SMT medical technology, Würzburg, Germany) and measured over a period of three days (Friday–Sunday). A day (24 h) was included in the analysis if the armband had been worn for at least 20 h and 30 min on at least two of the three days as described before [21]. The average wearing time per day in the present study was 98%. A measurement period of two days was included for 4 patients, three days for 57 patients.

The SenseWear™ armband is a wireless body monitor that enables continuous physiological measurement of PA outside the laboratory [22]. It consists of five sensors measuring heat flow, galvanic skin response, body temperature, near-body temperature and movements in two axes [23]. The two-axial accelerometer captures movements of the upper arm, as well as of the position of the body; with the help of the sensors, it is also possible to differentiate between movements such as locomotion in a vehicle or real PA, as well as to quantify physical inactivity such as time spent in a recumbent position [22]. In the current study, we focused on the following parameters of PA: (i) steps per day as an established and comprehensible parameter; (ii) time spent in a recumbent position including sleep time as an index of physical inactivity and (iii) duration of high intensity PA. The latter is quantified based on internal algorithms of the producer, and activity above 5.0 metabolic equivalents (METs) was considered as a high intensity exercise [21]. The MET value represents a standardized indicator independent of time, body weight and sex and mirrors the intensity of PA, ranging from 1.1 when driving in a car to 2–4 when doing housework; competitive athletes can reach maximum values of 20 METs [23]. A typical activity in the range of 5.0 METs is dancing (ballet, modern or jazz) [24].

### 2.7. Leptin Level Analysis

Blood samples were taken during the period of data collection for PA. All blood samples were collected from the forearm vein between 07:00 and 08:30 a.m. after an overnight fasting period of at least 10 hours as described previously [25]. Blood sampling took place within 4 ± 2 (1–11) days after admission to inpatient treatment. During venipuncture, blood was collected in pre-cooled standard laboratory EDTA tubes containing aprotinin (1.2 trypsin inhibitory unit per 1 mL blood; ICN Pharmaceuticals, Costa Mesa, CA, USA) for peptidase inhibition, placed back on ice immediately after blood withdrawal and centrifuged at 4 °C for 10 min at 3000× *g*. Plasma was separated and samples stored at −80 °C until further processing. The measurement of plasma leptin levels was conducted in one batch using a commercial enzyme-linked immunosorbent assay (#EK-003-17, Phoenix Pharmaceuticals, Inc., Burlingame, CA, USA). The linear detection range of the assay was 0.06–4 ng/mL (manufacturer’s information); the intra-assay variability was <10%.

### 2.8. Statistical Analysis

For statistical analysis, R Version 3.4.1 was used [26]. The distribution of data was determined using the Kolmogorov–Smirnov test. All data are presented as the mean ± SD if normally distributed, otherwise as the median (25th/75th percentile) or absolute frequency (relative frequency %). *p* < 0.05 was set for statistical significance. Leptin values were non-normally distributed. For linear modelling, leptin was log-transformed, but in the figures, the scale was not log-transformed to be able to display clinically-meaningful results. To test the relationship between PA and various potential predictors, univariate and multivariable linear models were computed. Graphical exploration of the data suggested non-linear relations. Accordingly, an exploratory regression tree was computed as this approach does not make assumptions about distributions or linearity. This machine learning technique computes a series of prediction thresholds to split a dataset. Given the relatively small sample, splitting the dataset into learning and test sets was not feasible; therefore, we applied a jack knife procedure classifying each subject based on a tree built from the remaining patients.

## 3. Results

### 3.1. Study Population

Altogether, 51 patients (84%) were diagnosed with typical and ten (16%) with atypical AN. All atypical AN patients were of the restrictive subtype. Twenty-three patients (36%) were smokers, and nine (15%) were on sedating medications on admission. On-body time recorded by the armband was 98 ± 2 (91–100)%. The demographic, anthropometric and disease-specific characteristics of the study population are outlined in Table 1 along with data on body composition and PA, as well as leptin levels. Duration of high intensity PA was recorded only for a median of two minutes per day. Thus, at least on average, PA in the form of high intensity exercise was negligible. All PA parameters assessed in this study showed a considerably wide range. Data on body composition were available in 60 of the 61 patients, and in 11 out of these 61 AN subjects (16.4%), BIA yielded a negative value for fat mass.

### 3.2. Relationship between Leptin, BMI, Body Composition and Physical Activity

**Figure 1:**
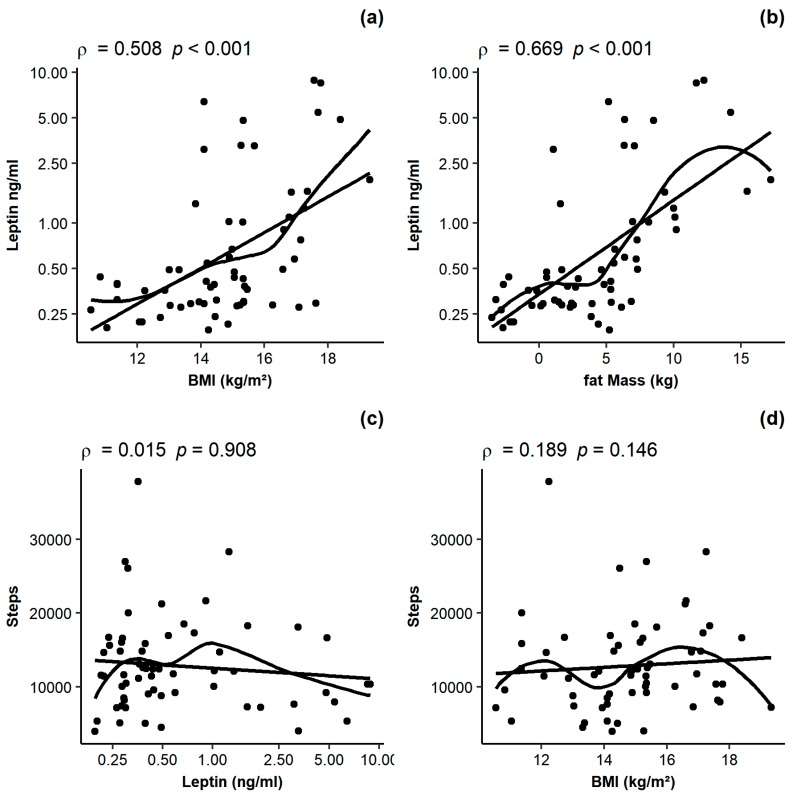
Associations between leptin and BMI (**a**), leptin and fat mass (**b**), steps and leptin (**c**) and steps and BMI (**d**).

Leptin was positively and highly associated with both BMI and fat mass (Figure 1a,b, ρ = 0.508 and ρ = 0.669, *p* < 0.001), but not with the step count (Figure 1c, ρ = 0.015, *p* = 0.908). In the whole population, no significant association of BMI and steps was observed (Figure 1d, ρ = 0.189, *p* = 0.146).

**Figure 2:**
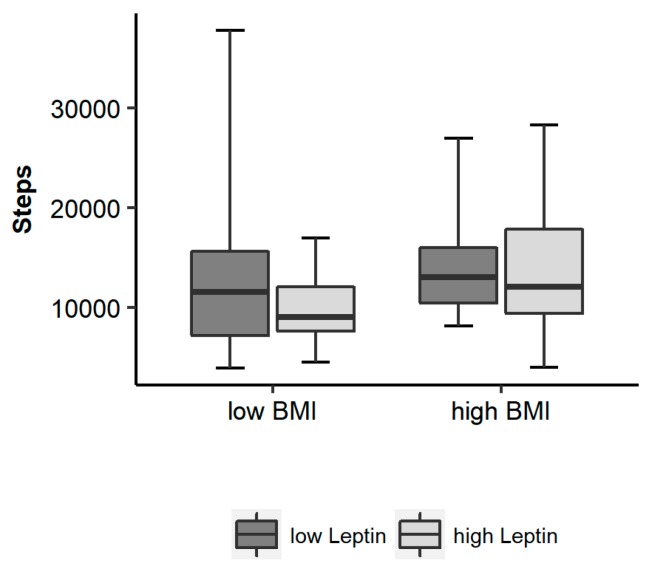
Steps in groups with high and low BMI and high and low leptin (median split).

When dividing the patients by two separate median splits into two groups of low and high leptin levels (median leptin = 0.41 ng/mL; group average with low leptin: 0.29; 25th/75th percentile: 0.26/0.36 ng/mL; group average with high leptin: 1.0; range: 0.5–3.2 ng/mL) and low and high BMI (median BMI = 14.9 kg/m^2^; group average with low BMI: 13.2 ± 1.3 kg/m^2^; group average with high BMI: 16.3 ± 1.2 kg/m^2^), respectively, a trend towards more steps was recorded in the group with higher BMI (*p* = 0.130), but not in the group with low leptin (*p* = 0.272, Figure 2).

### 3.3. Potential Moderators with an Effect on the Association of Leptin and Physical Activity

In a univariate regression analysis with steps as the dependent variable and age, BMI, recumbent time, duration of illness, sedating medication on admission, AN subtype and log leptin as independent predictor variables, only recumbent time was a significant moderator (*p* = 0.043). In a multivariable model, none of the independent variables remained as significant predictors.

**Figure 3:**
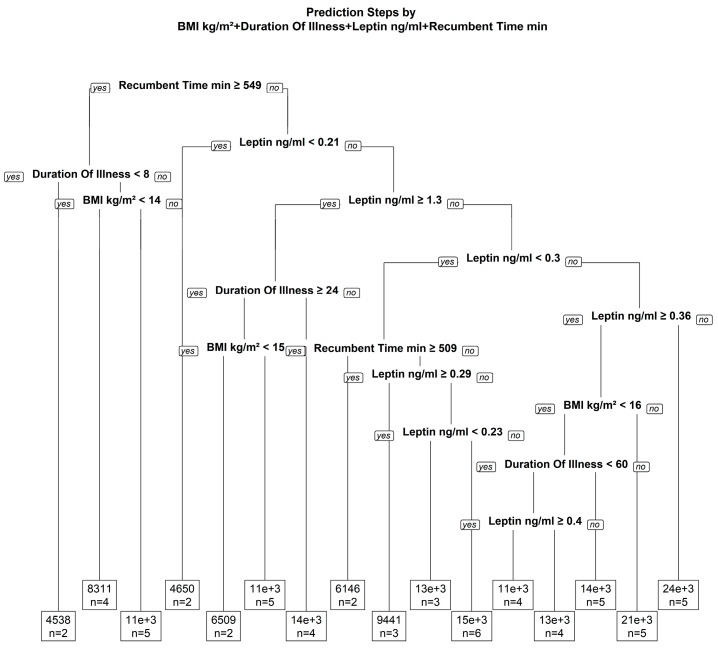
Regression tree for non-linear modelling to test the relation between physical activity and further parameters.

In an exploratory regression tree model, the following parameters were relevant predictors of the number of steps: BMI, duration of illness, leptin and recumbent time (Figure 3). With this non-linear model, the association of actual and predicted number of steps could be predicted with an *r*^2^ of 0.620 (*p* < 0.001). However, validation of prediction by jack knife analysis failed (*r*^2^ = 0.031; *p* = 0.176).

## 4. Discussion

This study analyzed the relationship between leptin and objectively assessed PA in a large group of adult, hospitalized women with AN. In the present study, there was a significant positive association of leptin and both BMI, as well as fat mass, as expected based on data in lean individuals [27]. However, no significant association was observed between low leptin levels and high PA, as reported earlier in studies on adolescents with AN [6,11,12,13]. In the adult population investigated in the current study, this association seemed to be non-linear and affected by a range of moderators and complex interactions. However, although the exploratory regression tree model, testing a non-linear model, yielded significant results and a high *r*^2^ of 0.62, these results were not replicated or significant when testing the robustness of the finding using a jack knife approach. This inconsistency could possibly be due to the small sample size or to overfitting of the original model.

With 14.8 kg/m^2^ as the mean and 10.5 kg/m^2^ as the minimum, the reference BMI of the patients in the present study was within the usual range reported in clinical studies [27]. Mean leptin levels of 1.2 ng/mL of our patients (although data were not normally distributed) were comparable to concentrations reported in previous studies (range: 0.9–5.6 ng/mL; [27]). Yet, it is to be noted that comparisons of absolute levels with other studies is difficult as different methods (ELISA, RIA) and kits of different vendors (Linco, Phoenix, DSL Systems, Millipore, Mediagnost), as well as lot-to-lot differences of the same kit might greatly influence the peptide levels measured. In line with these differences, the standard curves differ by factors up to 100. The linear detection range of the assay used in our study was 0.06–4 ng/mL, indicating that the mean leptin levels were in the middle of this curve.

Evaluating the degree of PA of the patients assessed in the present study in comparison with previous studies in AN is also complicated. PA at the start of hospitalization is increased in many, but not in all individuals with AN [28]. So far, most studies on PA have relied on nurses’ or therapists’ assessment. As patients’ self-reported PA did not correlate with measured PA, subjective PA assessment in AN should be interpreted with caution [10]. Few studies have objectively quantified increased PA in AN using accelerometry (Table 2). Some of these studies applied simple pedometers, which are limited in distinguishing whether the subject really was physically inactive or whether the pedometer was not worn, thus introducing a potential bias. This issue might be of particular importance in AN patients who are known to exercise in secret due to their fear of having their activity restricted. In a previous study using accelerometry in patients with AN, nine out of 51 measurements were not included in the analysis due to long periods of inactivity, which indicated Actiwatch™ misuse [14]. The advantage of the SenseWear™ armband is that data recording is initiated and stopped by contact of its heat flux sensor with the skin, enabling one to analyze on-body time and to discard measurements if the band were not worn.

AN, anorexia nervosa; BMI, body mass index; n.r., not reported; PA, physical activity; SIAB-EX, Structured Interview for Anorexic and Bulimic Eating [6,9,10,11,12,13,14]. Compared to normal weight or obese outpatients [21], the hospitalized AN patients in the present study were, with an average of 12,841 ± 6408 steps, far more physically active. We are aware of only two previous studies reporting on the number of steps assessed with the SenseWear™ armband in AN patients [29,30]. In one of these earlier studies, the number of steps recorded was similar between 53 hospitalized AN patients and non-hospitalized controls (AN: 8563 ± 5080; controls: 9638 ± 2983, *p* = 0.184) [29]. The other study measured PA at the end of 20 weeks of inpatient treatment in 32 weight-recovered AN patients who showed between 12,347 and 17,775 steps [30]. A commonly-used or accepted cut-off for high/low PA in AN does not (yet) exist. On average, during the three days of PA assessment, intensive exercise was recorded for just a few minutes in the patients under investigation, which is similar to a recorded time of 217 seconds of moderate and vigorous PA of between three and six METs in a previous study on adult and hospitalized AN patients [29]. The patients in the present study were allowed to move freely in the ward, as was the case in patients described in the study by El Ghoch and colleagues [29] (information available on author request). This is different from other treatment programs for AN patients, which commonly restrict any form of PA [28]. Therefore, for better interpretation and comparison between studies, future research on this topic should include information about whether or not PA was restricted as part of the treatment program. In both previous studies and in the present study using SenseWear™, AN patients displayed a high range of steps. Taken together, the patient sample in the present study displayed on average low leptin and high physical activity compared with previous studies, as well as high individual variability of activity levels within our group.

Leptin is a hormone predominantly secreted by adipocytes and circulating at levels proportionate to the amount of adipose tissue [31]. Thus, low leptin levels in AN can be explained by reduced body fat. Percent fat mass explains more variance in leptin levels than BMI [27]. Similarly, in the present study, the association was closer between leptin and fat mass than and BMI. Although not significantly associated in our study, also fat-free mass should always be considered, as the contribution of fat free-mass to BMI is higher in AN compared to normal weight subjects. Leptin might play a critical physiological role in the regulation of neuroendocrine systems during starvation, and low circulating levels are hypothesized to cause an increase in PA. However, the main finding of the present study was the lack of a simple and linear association of leptin and PA as measured by steps per day, which is in contrast to several previous studies. Table 2 [6,9,10,11,12,13,14] gives an overview of previous studies focusing on the relationship between leptin and PA in AN. Few studies have specifically reported on the association of leptin and PA in adult AN patients, showing both an association [12], as well as a lack thereof [10]. Hyperactivity related to hypoleptinemia in AN might be a complex phenomenon, as both neurobiological factors and deliberate attempts to lose weight by over-exercising seem to coexist [12]. Moreover, ethnicity affects leptin levels in lean and obese subjects [18,19]. Although the enrolment of Caucasian subjects can be assumed for all studies listed in Table 2, ethnicity was not specified in these studies. In the present study, only Caucasian subjects were included. Future studies should therefore investigate the alterations of leptin levels under the conditions of AN in different ethnic groups.

In addition, the relative contribution of these multiple underlying mechanisms is likely to differ according to the stage of the disorder [10,13]. In line with this view, the association of leptin and PA over time during the recovery from AN was shown to be dynamic; more specifically, a negative association at baseline changed into a positive correlation during the recovery process [10]. In the present study, there was a slight trend towards higher PA in patients with higher BMI, which might indicate that at a higher BMI, the patients’ health allows them to be more physically active. Moreover, age and duration of illness have been suggested as modulating factors of the association of leptin and PA [10]. The duration of illness was significantly higher in highly active patients than in patients with low activity, and highly active patients had an earlier age of illness onset [14]. In theory, the time course could be as follows: at an early stage of starvation, neurobiological factors, as reflected by decreasing leptin levels, may be of importance to increase food-searching behavior [12]. The resulting “agitated restlessness” might persist during the early course of the disease, but at some point, no longer apply if starvation persists. This shift might be due to an adaptation to low peripheral leptin levels in chronically ill and older patients, with their activity levels no longer being influenced by leptin [13]. When starvation progresses even further, formerly increased activity could turn into lethargy and weakness at very low BMI in the later stages of starvation [10].

Currently, the findings from different studies on the relationship between leptin and PA are inconsistent. While our study cannot resolve these existing controversies, we took a first step towards dissecting the influence of various moderators on the association of leptin and PA in AN. Exploratory analyses applying a complex and non-linear model including potential moderators and interactions yielded promising results for the following moderators: age, BMI, duration of disease, AN subtype and intake of sedating medication. Nevertheless, as mentioned above, the small sample size for such analyses and the potential for overfitting limit the conclusions that can be drawn from these initial findings.

A differentiated understanding of increased PA as a fundamental symptom of AN has the potential to improve therapeutic options. First, in some patients and treatment phases, increased PA seems to be a neurobiological consequence of starvation, rather than being a conscious attempt of patients to lose weight or representing noncompliance with treatment. Second, the administration of leptin in an acute state of illness to reduce “uncontrollable” hyperactivity has been suggested for the treatment of humans [13,28], provided that specific precautions are taken. Evidently, the metabolic and nutritional situation of the leptin-treated patient needs to be monitored closely due to the possible increase in resting energy expenditure and energy needs. For a long time, leptin administration in clinical human trials seemed unlikely. Recently, leptin administration received approval for the treatment of lipodystrophy [31], making it more likely that leptin administration in patients with AN could follow in the near future. According to the data presented here, an evidence-based procedure should first be developed to identify individual patients and treatment phases where leptin administration can be beneficial. Patients exercising mainly to pursue thinness might be less likely to benefit from this treatment. Therefore, next to biological determinants of PA described in our study, clinical and research assessment should also include eating disorder-specific psychopathology related to hyperactivity.

There was a significant association of recumbent time and number of steps (*p* = 0.043) in our study population. Whether a low number of steps results invariably from the fact that during recumbent time no steps can be taken, or whether high recumbent time could be a sign of physical weakness or serve as a marker of low motor restlessness remains unclear. We are not aware of any studies that objectively assessed sedentary behavior in patients with AN, which therefore remains to be uncovered further. In obesity, adverse metabolic effects of sedentary behavior have been established [32], and therapeutic options target the reduction of sedentary time to improve cardiometabolic health [33].

The findings of this study need to be interpreted within its limitations. First, with 61 subjects, the samples size of this study was still too small for valid subgroup analyses to build a sound, non-linear model, which should be attempted in future studies. Second, we focused on the assessment of steps as a marker for PA. However, it seems likely that hyperactivity in AN can manifest in several forms [14], such as high intensity exercise, which was negligible in the present study, or a more subtle, but constant “motor restlessness”. While there is the need to assess PA using objective techniques [13], it is remarkable that the present and the only other study that used accelerometry [14] did not find an association of PA and leptin. Inconsistent results regarding the association of leptin and PA in AN could therefore be of methodological origin. Judged from the present study, it can further be questioned if the number of steps per day is a suitable marker for altered PA in AN. However, another study provided evidence that the number of steps assessed at the end of treatment predicted the resumption of menses at one-year follow-up in patients with AN [30], pointing towards the utility of steps as a clinical-biological marker. Further work is needed to determine how motor restlessness (as opposed to PA) can best be measured and quantified with accelerometric devices. Third, we allowed the patients to take off the band for a maximum of 2.5 h/day, i.e., to take a shower or in the case of feeling discomfort by the elastic band. While the average wearing time was 98% (range: 91–100%) of the total time, we cannot exclude the possibility that during part of or the entire remaining 2% (range: 0–9%), the patients could have been highly active. On the other hand, we consider the technology used in the present study to be the best available method due to its heat sensors and the ability to detect how long the band was actually worn. Fourth, we used BIA to assess body fat, which is an easy to use technique, but its accuracy in patients with AN is reduced compared to the general population [34]. Finally, the present study was limited by the fact that it did not include data from a healthy control group. However, usually healthy controls cannot be assessed under the same conditions in a hospital setting and subjected to an eating disorder program, which makes a comparison of PA in patients versus healthy controls difficult. However, a control group should be included in future studies using an outpatient setting.

## 5. Conclusions

In conclusion, the association of leptin and physical activity in adult females with AN in the present study was not of a simple, linear nature and requires further investigation with respect to its potential modifiers, including age, BMI, duration of illness and medication, which may operate in a non-linear fashion or differ across subgroups. Moreover, behavioral and psychological factors should be psychometrically assessed in detail and taken into consideration, especially in light of common psychiatric comorbidities [35]. Lastly, the most valid use and interpretation of accelerometry employed to objectively assess PA in AN patients needs to be established further and should also include different parameters of physical inactivity.

## Figures and Tables

**Table 1:** Characterization of the study population.

Measurement Parameters	All Patients	Restrictive Subtype	Purging Subtype	*p*-Value
*n* (%)	61 (100%)	49 (80.3)	12 (19.7)	
Demographic parameters				
Age (years)	25 (21/30; 18–52)	24 (19/32; 18–52)	26 (21/29; 18–32)	0.643
Height (m)	1.67 ± 0.07 (1.53–1.84)	1.67 ± 0.07 (1.53–1.84)	1.69 ± 0.06 (1.55–1.80)	0.233
Weight (kg)	41 ± 7 (29–59)	41 ± 7 (29–59)	45 ± 4 (37–49)	0.056
BMI (kg/m^2^)	14.8 ± 2.0 (10.5–19.3)	14.6 ± 2.1 (10.5–19.3)	15.7 ± 1.6 (13.3–18.4)	0.085
Duration of illness (months)	44 (12/120; 1–312)	33 (12/77; 1–312)	84 (25/120; 4–180)	0.243
Body composition				
Fat-free mass (kg)	37 ± 4 (30–48)	37 ± 4 (30–48)	40 ± 4 (32–48)	0.016
Fat mass (kg)	4.3 ± 4.7 (−3.5–17.2)	4.1 ± 5.2 (−3.5–17.2)	4.9 ± 2.3 (0.6–7.3)	0.599
Fat mass (%)	9.1 ± 10.2 (−10.7–29.5)	8.6 ± 11.2 (−10.7–29.5)	11.0 ± 5.1 (1.2–15.3)	0.464
Physical activity				
Steps	12,841 ± 6408 (3956–37,750)	13,069 ± 6717 (3956–37,750)	11,908 ± 5091 (4534–18,505)	0.578
Recumbent time (min)	480 (445/533; 333–666)	473 (443/527; 333–581)	502 (464/569; 407–666)	0.174
Leptin (ng/mL)	0.4 (0.3/1.0; 0.2–8.8)	0.4 (0.3/1.0; 0.2–8.8)	0.5 (0.3/2.1; 0.3–4.9)	0.246
Log leptin (ng/mL)	−0.46 ± 1.04 (−1.62–2.19)	−0.51 ± 1.05 (−1.62–2.19)	−0.27 ± 1.01 (−1.27–1.59)	0.480

Mean ± SD (range) or median (25th/75th percentile; range); bolded *p*-values: *p* < 0.05.

**Table 2:** Previous studies reporting on the association of leptin and physical activity in patients with AN.

Reference	Subjects (*n*)	Mean Age (Year) (Range)	Mean BMI (kg/m^2^) (Range)	Mean Leptin (ng/mL) (Range)	Assessment of Physical Activity	Association Leptin-Physical Activity	Medication	Physical Activity Restriction
Exner 2000	30	16.4 ± 3.5 (12–31)	14.5 ± 1.4 (11.8–16.8)	0.65 ± 0.56 (0.02–1.62)	patient ratings of motor restlessness	upon attainment of maximal leptin; motor restlessness was ranked lower compared to baseline	n.r.	n.r
Holtkamp 2003	61	17.5 ± 4.6 (12–31)	14.5 ± 1.5 (10.5–17.4)	0.91 ± 1.37 (0.004–7.43)	self and expert rating	association of expert rating of motor restlessness and leptin	no	n.r.
	27	14.5 ± 1.3 (11.5–17.4)	14.5 ± 1.3 (12.3–17.5)	1.77 ± 1.06 (0.53–4.57)	SIAB-EX interview	association of expert rating of motor restlessness and leptin; leptin, but not BMI, explained the variance of PA	n.r.	
Holtkamp 2006	26	15.6 ± 1.9	15.2 ± 1.6	1.3 ± 0.76	SIAB-EX interview	leptin predicted all types of activity and restlessness	n.r.	n.r.
Van Elburg 2007	31 31	15.9 ±1.2 20.6 ± 2.9	15.4 ± 1.3	2.1 (0.5–13.3)	nurse evaluation	on admission: linear association between leptin/PA in adolescents, but not in adults	57% on antipsychotics	n.r.
Ehrlich 2009	36	18.2 ± 3.3 (14–29)	15.3 ± 1.3	1.4 ± 2.3	SIAB-EX interview	inverse association in acute. but not in recovered AN; when samples were split according to age (22 adolescents, 14 adults): linear association remained significant in both groups	n.r.	
Nogueira 2010	24	22.8 ± 5.9	13.5 ± 1.2	0.75 ± 1.0	SIAB-EX interview	leptin levels higher in high level PA patients	yes	n.r.
Kostrzewa 2013	37	15.2 (13–17.5)	15.7 ± 1.4 (12.6–18.4)	n.r.	accelerometry	no significant difference in leptin between high and low PA patients	n.r.	yes
Haas 2017	61	25 (18–52)	14.8 (10.5–19.3)	1.2	accelerometry	no association of leptin and steps	15%	no

## Abbreviations

AN	anorexia nervosa
BMI	body mass index
PA	physical activity
SD	standard deviation
